# Visualising spatio-temporal distributions of assimilated carbon translocation and release in root systems of leguminous plants

**DOI:** 10.1038/s41598-020-65668-9

**Published:** 2020-06-11

**Authors:** Yong-Gen Yin, Nobuo Suzui, Keisuke Kurita, Yuta Miyoshi, Yusuke Unno, Shu Fujimaki, Takuji Nakamura, Takuro Shinano, Naoki Kawachi

**Affiliations:** 1 Takasaki Advanced Radiation Research Institute, National Institutes for Quantum and Radiological Science and Technology (QST), Gunma, 370-1292 Japan; 2 Department of Radioecology, Institute for Environmental Sciences, Aomori, 039-3212 Japan; 3 Agro-environmental Research Division, NARO Hokkaido Agricultural Research Center, Hokkaido, 062-8555 Japan; 4 Agricultural Radiation Research Center, NARO Tohoku Agricultural Research Center, Fukushima, 960-2156 Japan; 5 Present Address: Materials Sciences Research Center, Japan Atomic Energy Agency, Tokai, Ibaraki 319-1195 Japan; 6 Present Address: Institute for Quantum Life Science, National Institutes for Quantum and Radiological Science and Technology, Chiba, 263-8555 Japan; 7 Present Address: Research Faculty of Agriculture, Hokkaido University, Sapporo, Hokkaido 060-8589 Japan

**Keywords:** Positron-emission tomography, Plant physiology

## Abstract

The release of rhizodeposits differs depending on the root position and is closely related to the assimilated carbon (C) supply. Therefore, quantifying the C partitioning over a short period may provide crucial information for clarifying root–soil carbon metabolism. A non-invasive method for visualising the translocation of recently assimilated C into the root system inside the rhizobox was established using ^11^CO_2_ labelling and the positron-emitting tracer imaging system. The spatial distribution of recent ^11^C-photoassimilates translocated and released in the root system and soil were visualised for white lupin (*Lupinus albus*) and soybean (*Glycine max*). The inputs of the recently assimilated C in the entire root that were released into the soil were approximately 0.3%–2.9% for white lupin within 90 min and 0.9%–2.3% for soybean within 65 min, with no significant differences between the two plant species; however, the recently assimilated C of lupin was released at high concentrations in specific areas (hotspots), whereas that of soybean was released uniformly in the soil. Our method enabled the quantification of the spatial C allocations in roots and soil, which may help to elucidate the relationship between C metabolism and nutrient cycling at specific locations of the root–soil system in response to environmental conditions over relatively short periods.

## Introduction

The allocation of limited photoassimilates into the belowground plant parts is influenced by the various factors affecting the root–soil system, including root elongation, carbon (C) release, adaptation to the surrounding soil environment, and energy-requiring ion solubilisation and absorption^1–6^. Plant roots release a diverse variety of C compounds, termed rhizodeposits, into the rhizosphere, which extends several millimetres from the roots into the surrounding soil. It is estimated that approximately 3% of the assimilated C, corresponding to about 50% of the root biomass, is released by crops as rhizodeposits that remained in the soil^7^, including several organic compounds, border cells, and root exudates^4,5,8^. The release of organic substances occurs at a considerable cost to plants and, therefore, must be strictly regulated^3,4^. However, such regulatory mechanisms have not been fully elucidated owing to experimental technical difficulties.

In previous studies, various experimental approaches were developed to determine and estimate the C partitioning in plant roots and rhizosphere soil. Most of the commonly used approaches involve the destructive sampling of roots and rhizosphere soil. However, non-invasive approaches have also been developed to sample the soil solution near the roots^5,9^. These methods are useful for sampling the integrated amounts of released rhizodeposition sets at daily or weekly intervals during the plant growth period. However, the time lag between photosynthesis and soil CO_2_ efflux ranged from about a few hours to a few days, depending on the plant species (grasses or trees) and environmental conditions, including temperature^10,11^. A previous study proved that photoassimilates are translocated into the belowground plant parts very quickly and the rhizodeposits and root-derived CO_2_ from crops and grasses are detectable within the first few hours^7^. Additionally, photoassimilates are also very rapidly translocated into soybean roots (i.e., within 20 min)^12,13^. Therefore, C may also be quickly and continuously released^14^. The C allocation pattern in the belowground area of a rice paddy system was strongly dependent on the chase period and plant age, and the level of C markedly decreased in both roots and soil as plant growth proceeded^15^. Overall, some photoassimilates released as root exudates in the rhizosphere may exhibit pronounced short-term dynamics and have been suggested to be closely associated with root development. Thus, integrated and/or long-term sampling methods may not be the most appropriate for determining the detailed functions of the partitioning of such photoassimilates. A method that enables the quantitative analysis of short-term photoassimilate transport into the root and the surrounding soil with positional information is desirable for clarifying the C release capabilities at specific root positions.

Experimental systems based on pulse-labelling of ^14^C or ^13^C are commonly used to analyse photoassimilate allocation in the roots and rhizosphere^7,14–18^. In particular, imaging methods based on pulse-labelling of ^14^C have been applied to visualise the allocation of photoassimilates in the roots and surrounding area as rhizodeposits. For example, plant roots containing ^14^C-labelled photoassimilates translocated from the leaves to the roots as well as ^14^C-rhizodeposits released onto moistened filter paper have been visualised using autoradiography^19–23^. Additionally, soil-grown roots have been used to visualise C partitioning in the root and soil after several hours (or days) of ^14^CO_2_ pulse labelling^18,24^. However, a disadvantage of these experimental systems is that it is difficult to noninvasively visualise and quantify the distribution of C in root systems and the surrounding soil, because some of the rhizodeposits penetrate the soil surface by diffusion, and the beta-rays from ^14^C inside the root system and surrounding soil cannot penetrate the soil to be detected.

The positron-emitting tracer imaging system (PETIS) is an advanced imaging method for the quantitative analysis of radiotracer movements in intact plant bodies^13,25–27^. This method shares detection principles with positron emission tomography, which is mainly used in medical fields. The transport of ^11^C-labelled photoassimilates (^11^C-photoassimilates) from leaves to sink organs (e.g., roots and fruits) has been visualised previously using the PETIS^13,27,28^ or by positron emission tomography in combination with magnetic resonance imaging^29,30^ after pulse-feeding ^11^CO_2_ to plant leaves. In particular, the PETIS allows the visualisation of C translocation into the soil-grown root system because of the high energy of the gamma-rays (511 keV) from ^11^C that can permeate the soil^13,28^. Thus, it can be detected noninvasively. However, to the best of our knowledge, ^11^C-based imaging of photoassimilates in the surrounding soil coupled with the imaging of photoassimilate transport into the root system has not been reported. This may have been because of technical difficulties in distinguishing a small amount of ^11^C-photoassimilates in the surrounding soil from the much larger amounts of ^11^C-photoassimilates in the adjacent roots.

In this study, we hypothesised that the distributions of C transported to the whole root system through the phloem and released to the surrounding soil may differ depending on the positions of the root systems and the plant species. Therefore, the objective of this study was to develop a new method to visualise and evaluate the movement of ^11^C-photoassimilates into the root system and the release of ^11^C-photoassimilates into the soil coupled with positional information using the PETIS. The experiments allowed the characterisation of the different spatial C distributions in the rhizobox soil of white lupin (*Lupinus albus*) and soybean (*Glycine max*) plants.

## Methods

### Rhizobox design for imaging

**Figure 1:**
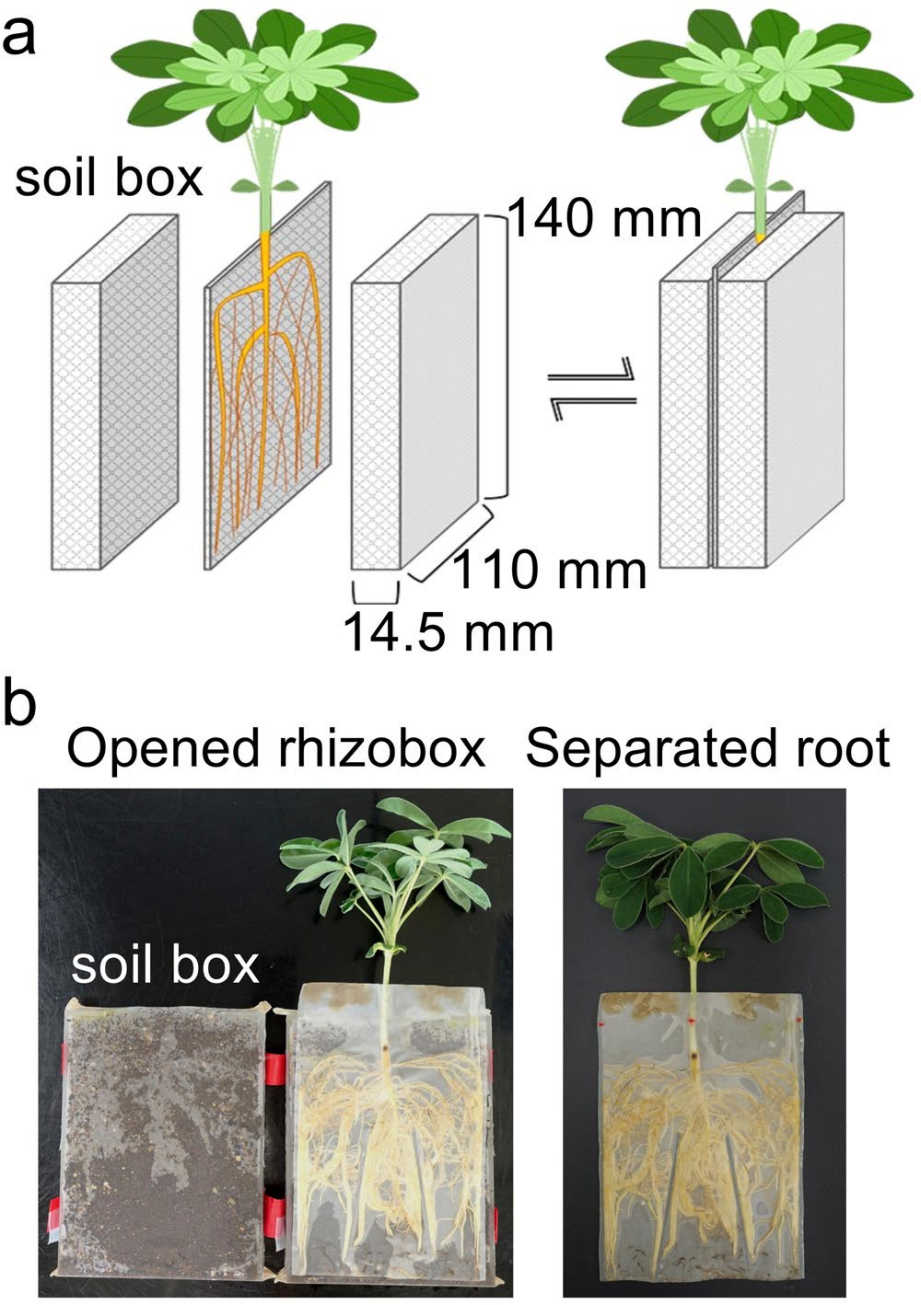
Configuration of a new rhizobox designed for the imaging experiments. (**a**) Schematic illustration of the rhizobox. A square nylon-mesh bag enclosing the root system is placed between two soil-containing boxes that are each covered with fitted nylon-mesh bags. The root system is sandwiched between the two soil boxes during the cultivation. (**b**) Plant growing in the rhizobox (left) and the root system with the nylon-mesh bag after removal from the rhizobox (right).

To distinguish the ^11^C signals of the soil from that of roots, a rhizobox was specifically designed for the PETIS imaging experiments. The assembled rhizobox consisted of a square nylon 48-μm mesh (Clever Co. Ltd, Aichi, Japan) bag and a pair of soil boxes (Fig. 1a). Each soil box was 140-mm high × 110-mm wide × 14.5-mm deep. The soil box was filled with 170 g dry commercial garden soil (pH 5.7) consisting of red soil, compost, and fertiliser (0.48 mg g^−1^ nitrogen, 1.56 mg g^−1^ phosphorus, and 0.48 mg g^−1^ potassium) (Flower and Vegetable Soil; Tachikawa Heiwa Nouen Co. Ltd, Tochigi, Japan). The soil box was covered with a fitted nylon-mesh bag to confine the soil to the box. The nylon-mesh bag was created by sealing the nylon mesh using an electrothermal sealer (HAKKO Corporation, Osaka, Japan). Numerous 5-mm-diameter holes in the base of the soil box allowed water absorption.

### Plant materials

The test plants were placed in a growth chamber maintained at 12 hours light (26 °C):12 hours dark (20 °C), with a photon flux density of 650 µmol m^−2^ s^−1^ at the tops of plants and 65% relative humidity for the entire cultivation period.

Seeds of white lupin (*Lupinus albus* L. ‘Energy’) were sterilised with 70% ethanol for 5 min and then thoroughly washed with deionised water. The seeds were sown on a vermiculite bed and then incubated in a growth chamber at 26 °C in the dark. One week after sowing, the seedlings were transferred to plastic containers holding 6 l of the following nutrient solution: 115.03 mg l^−1^ NH_4_H_2_PO_4_, 53.49 mg l^−1^ NH_4_Cl, 109 mg l^−1^ K_2_SO_4_, 0.935 mg l^−1^ KCl, 294.0 mg l^−1^ Ca(NO_3_)_2_·4H_2_O, 123 mg l^−1^ MgSO_4_·7H_2_O, 0.367 mg l^−1^ H_3_BO_4_, 0.302 mg l^−1^ MnSO_4_·5H_2_O, 0.004 mg l^−1^ (NH_4_)_6_Mo_7_O_24_, 0.081 mg l^−1^ ZnSO_4_, 0.032 mg l^−1^ CuSO_4_·5H_2_O, 0.051 mg l^−1^ CoSO_4_·7H_2_O, 0.004 mg l^−1^ NiSO_4_·6H_2_O, 18.6 mg l^−1^ ethylenediamine-*N*,*N*,*N*′,*N*′-tetraacetic acid disodium salt dihydrate, and 13.9 mg l^−1^ FeSO_4_·7H_2_O at pH 5.2. The nutrient solution was renewed every 4 days during the 10 days hydroponic cultivation period.

Seeds of soybean [*Glycine max* (L.) Merr. ‘Jack’] were sterilised with 70% ethanol for 30 s and with 5% sodium hypochlorite solution for 20 min, and then thoroughly washed with deionised water. To reproduce the normal soybean roots grown in the field, the seeds were inoculated with a suspension of *Bradyrhizobium diazoefficiens* strain USDA110 for 2 hours, sown on a vermiculite bed, and then incubated in a growth chamber at 26 °C under dark conditions. One week after sowing, the seedlings were transferred to plastic containers holding 6 l of the nutrient solution^31^. The nutrient solution was renewed every 7 days during the 18 days hydroponic cultivation period.

After hydroponic cultivation, each root system of the white lupin and soybean plants was placed into a square nylon-mesh bag and sandwiched between two soil boxes. The plants were grown for another 10 days in the same growth chamber with the same conditions. The root systems could absorb water and nutrients from the soil through the mesh. The root system was easily removed from the two soil boxes without disturbing the soil surface (Fig. 1b). Water was initially added from the top of the rhizobox to produce a depth of approximately 3 cm in the watering tray every three days. Then the water was supplied through the holes at the bottom of the rhizobox. The holes were covered with nylon mesh to prevent lessivage.

### PETIS imaging experiment

#### ^11^CO_2_ tracer gas

The ^11^CO_2_ was produced by the reaction of ^14^N(*p,α*)^11^C caused by bombarding pure nitrogen gas with 10 MeV protons from an azimuthally varying field cyclotron located at the Takasaki Ion Accelerators for Advanced Radiation Application, National Institutes for Quantum and Radiological Science and Technology (Gunma, Japan)^28^. The irradiated gas that contained the nitrogen gas and ^11^CO_2_ was passed through a stainless-steel pipe cooled with liquid nitrogen. Only the ^11^CO_2_ gas was collected as dry ice in the stainless-steel pipe; its radioactivity was measured with a curimeter (IGC-3, Aloka, Tokyo, Japan). The resultant 190–450 MBq of the ^11^CO_2_ (corresponding to approximately 0.56–1.32 pmol) was transferred to the gas conditioning system for the PETIS imaging experiment.

#### PETIS measurement

**Figure 2:**
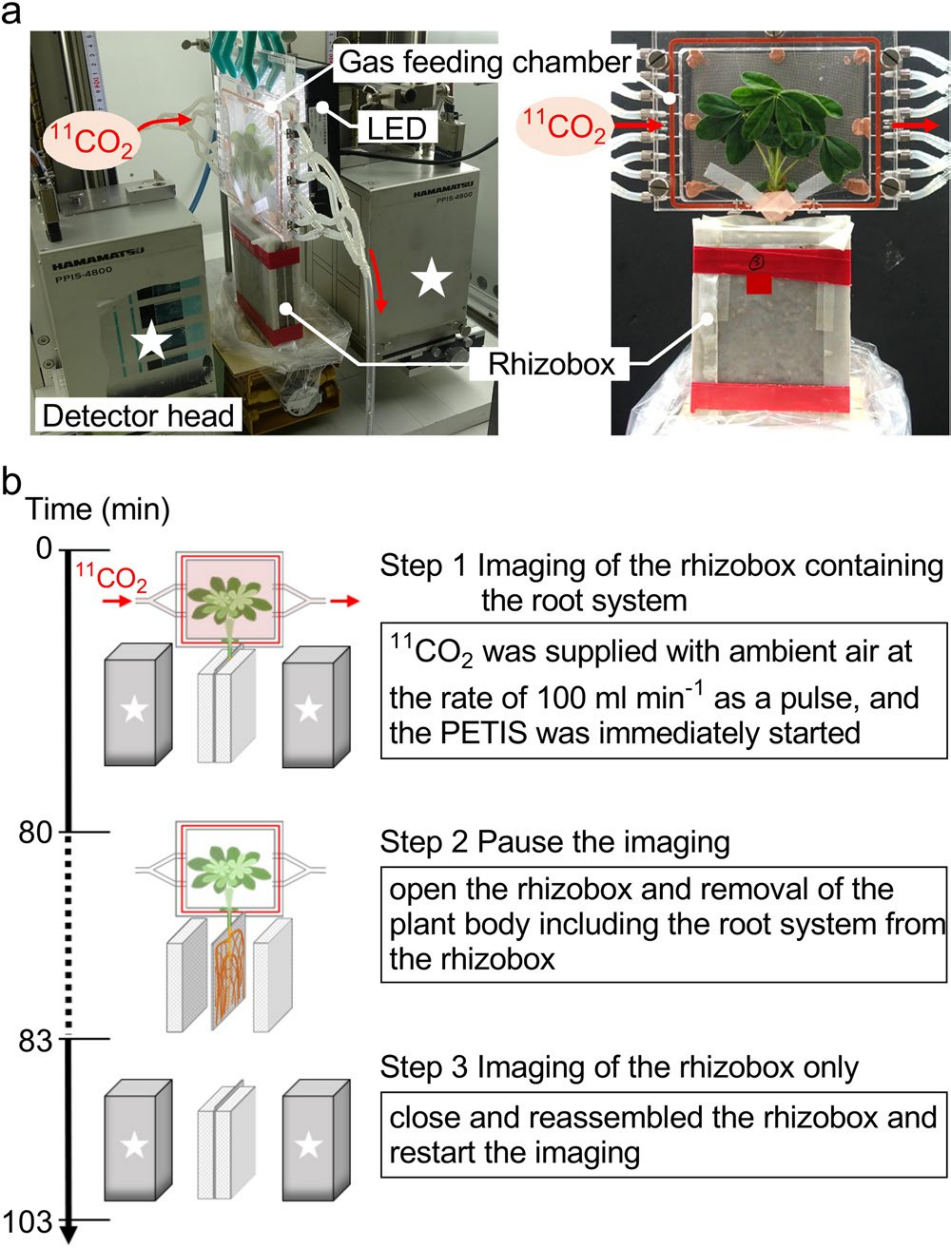
Equipment and protocol for the imaging experiment. (**a**) Placement of the test plant, gas feeding chamber, and light-emitting diode (LED) during the positron-emitting tracer imaging system (PETIS) experiment. (**b**) Procedure for the PETIS imaging experiments.

The PETIS was installed in the growth chamber during the imaging experiment (Fig. 2a). The PETIS apparatus, a modified PPIS-4800 (Hamamatsu Photonics K.K., Hamamatsu, Japan), had a field of view (FOV) that was 119.9-mm wide and 187.0-mm high, with a spatial resolution of 1.6–2.1 mm for the entire FOV^32^.

The rhizobox area of each test plant (four lupin and four soybean plants) was positioned just inside the FOV at the focal plane between two opposing PETIS detector heads. The photon flux density was maintained at 1,000 µmol m^−2^ s^−1^ throughout the imaging experiments. The leaves of the test plant were covered by a gas-tight feeding chamber to supply the ^11^CO_2_ tracer gas. The collected ^11^CO_2_ was extruded by a pump and mixed with ambient air (containing approximately 450 ppm CO_2_) supplied in one direction from the inlet to the outlet of the feeding chamber. The mixed tracer gas was supplied at a constant rate of 100 ml min^−1^, and the PETIS was immediately started to obtain images (Fig. 2b, Step 1). The ^11^CO_2_ was passed through the feeding chamber within 1 min and the residual ^11^CO_2_ that was not fixed by leaves was completely collected by soda lime (Wako Pure Chemical Industries Ltd., Osaka, Japan) connected to the outlet of the feeding chamber. The movement of ^11^C-photoassimilates to the root system was monitored in real-time. When the ^11^C signal reached the root tips, at 70–90 min for white lupin and 65 min for soybean after the ^11^CO_2_ feeding, the whole plant with intact roots was quickly removed from the rhizobox (Fig. 2b, Step 2). Then, the rhizobox was reassembled, and the PETIS imaging resumed to visualise the released ^11^C present in the rhizobox soil without plant roots. The imaging continued for 20 min (Fig. 2b, Step 3). Images were acquired every 10 s.

An additional PETIS experiment was conducted to analyse the kinetics of ^11^C-photoassimilates translocated into the root and ^11^C-labelled organic substances released by the root into a hydroponic nutrient solution in a 35 days old soybean plant (Fig. S3a). After the ^11^CO_2_ feeding, the nutrient solution was continuously circulated by a pump (UPS-112E, NITTO KOHKI CO., LTD., Tokyo, Japan) at 13 ml min^−1^ through an InertSep Active Carbon column cartridge (GL Sciences Inc., Tokyo, Japan) to collect the released ^11^C-organic substances. Time-course analyses of ^11^C-labelled photoassimilates in the roots and cartridge were generated from the PETIS imaging data (Fig. S3b).

The prepared PETIS images were corrected for the sensitivity distribution and the counting efficiency of the gamma-rays on the detectors by using a standard radiation source, and for ^11^C radioactive decay (T_1/2_ = 1,223.1 s)^29^. In this way, we ensured that the PETIS images were quantitative with respect to the ^11^C radioactivity.

### Analyses of the PETIS data

**Figure 3:**
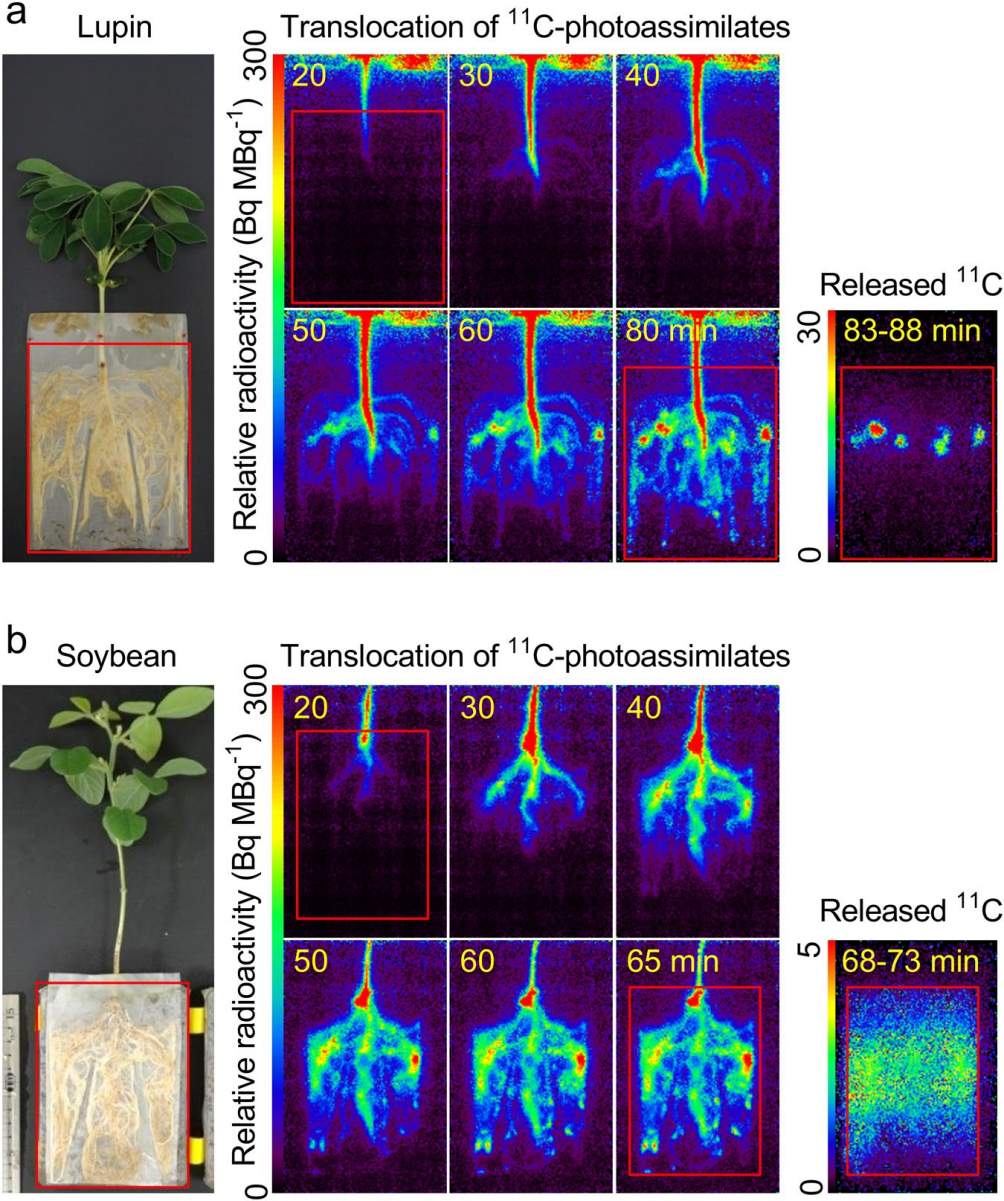
Imaging data of ^11^C-photoassimilate distributions in the root system and the rhizobox soil for one lupin plant and one soybean plant. Images of the plant with a root system (a and b, left panel) and serial images of ^11^C-photoassimilates in the root system after feeding with a ^11^CO_2_ tracer gas (a and b, middle panel). Each image is a composite of original images collected at 1-min intervals. Serial images of released ^11^C in the soil after removal of the plant body from the rhizobox (a and b, right panel). Each image is a composite of original images collected at 5-min intervals. All images were corrected for ^11^C radioactive decay. The red square indicates the area of the rhizobox in the field of view.

**Figure 4:**
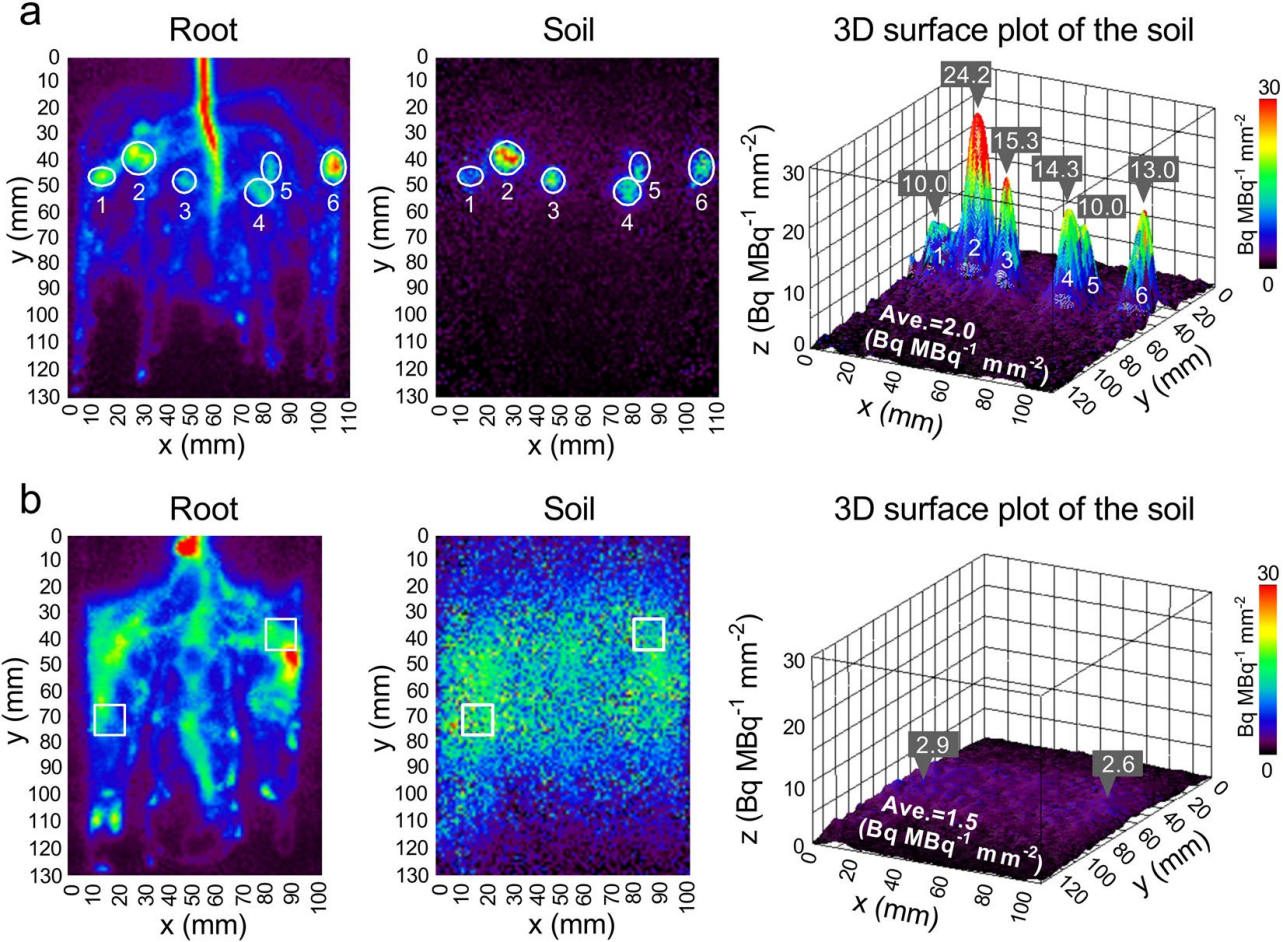
Regions of interest (ROIs) and 3-D surface plots of the rhizobox soil with white lupin (**a**) and soybean (**b**) from Fig. 3. The ROIs were set in the imaging data of both the root and soil of white lupin (circle) and soybean (square). The imaging data of soil was used to generate a 3-D surface plot, with the *z*-axis indicating the ^11^C radioactivity levels (Bq). The value of each ROI indicates the density of relative ^11^C radioactivity in the area (mm^2^) of each ROI. The results of the other six test plants are shown in Fig. S2.

**Figure 5:**
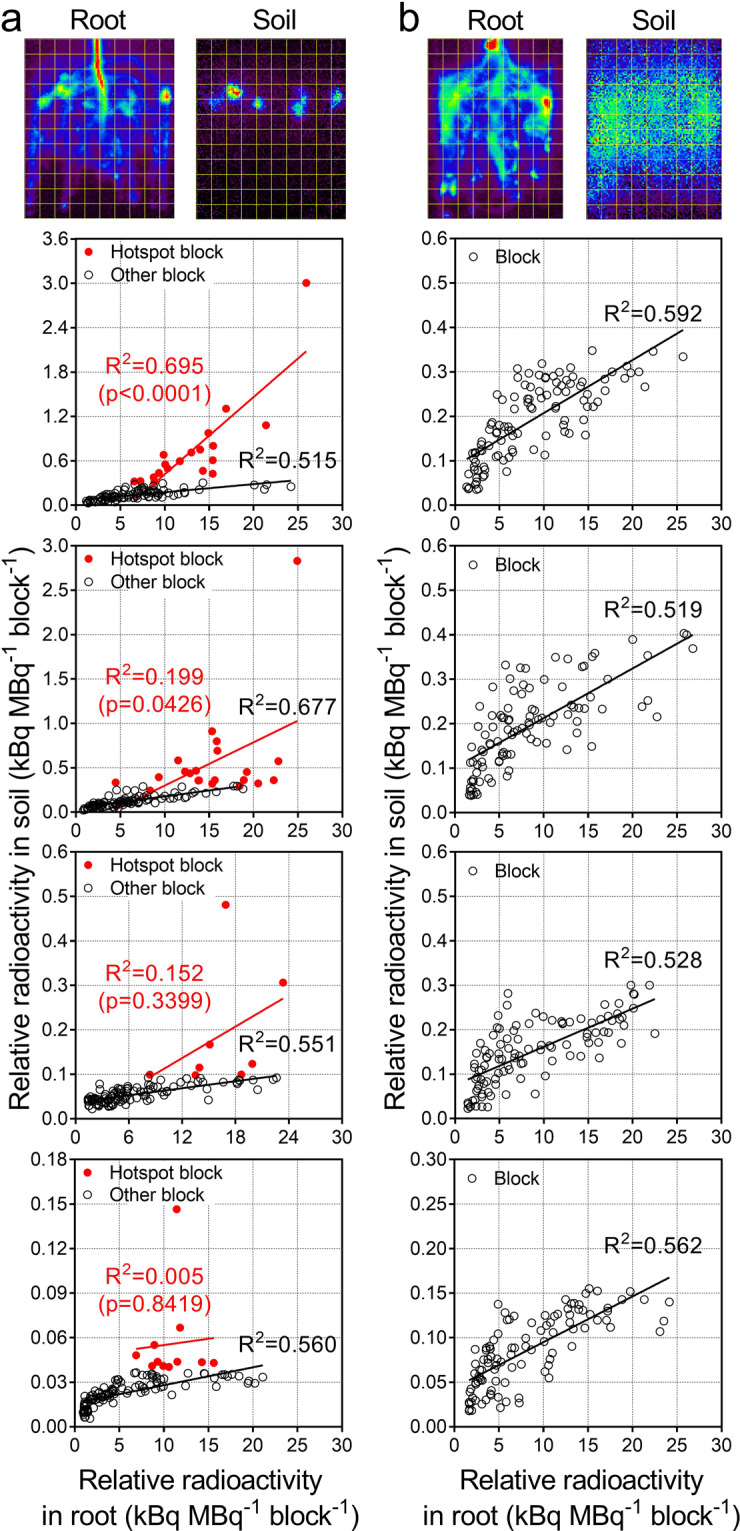
Scatter plots between the relative ^11^C radioactivity of the root and soil in four test plants of white lupin (**a**) and soybean (**b**). Each plot indicates the relative ^11^C radioactivity value of each block and corresponds to the 11-mm × 11-mm square ROIs in the images at the top of the figures of white lupin and soybean. The hotspot block (red circles) was selected to include the entire defined hotspot area presented in Figs. 4a and S2a. The other block (white circles) did not include the hotspot area. For white lupin, the coefficient of determination (R^2^) values were calculated for the hotspot block and the other block. For soybean, all blocks were used to calculate the R^2^.

Serial images of the ^11^C-photoassimilates moving through the root system and the released ^11^C in the rhizobox soil were obtained (Figs. 3a,b and S1). The original PETIS image data were acquired as counts of gamma-rays at 10 s per frame. The counts of every six frames of image data were averaged and integrated to yield data at 60 s per frame for the root system. The same method was used to obtain data at 5 min per frame from every 30 frames of the original image data for released ^11^C into the soil. The PETIS imaging data were prepared using ImageJ ver. 1.51J8 software (https://imagej.nih.gov/ij/). To align the image data intensity, all imaging data were normalized to the total arrived ^11^C radioactivity in the entire underground part of the rhizobox area within the root imaging period, i.e., including the ^11^C radioactivity of the roots and soil (Figs. 3 and S1). The value of total arrived ^11^C radioactivity was calculated from the average ^11^C radioactivity in a region of interest (ROI) set on the rhizobox area during the last 5 min of the root imaging period for each of the eight plants (four lupin and four soybean plants). Therefore, the colour scale of the imaging data of Figs. 3 and S1, the *z*-axis of Figs. 4 and S2, and the values in Fig. 5 indicate the relative ^11^C radioactivity levels.

On the basis of the PETIS imaging data and the superimposition onto the pictures taken after the imaging experiment, some ROIs were selected in the imaging data of both the root and rhizobox soil (Figs. 4 and S2). The ROIs were set by enclosing the high signal intensity regions selected in visual with different sized circles as the hotspot for the soil and for the corresponding positions on the roots in lupin (Figs. 4a and S2a). Two representative square ROIs were set on the high signal intensity regions of both sides of the soil and roots in soybean (Figs. 4b and S2b). The imaging data for the soil was used to generate a 3-D surface plot to indicate the relative ^11^C radioactivity levels (*z*-axis). Because the ROIs did not have the same area, to align and compare the image intensity for the different ROIs, the relative ^11^C radioactivity per unit area (mm^2^) of each ROI was calculated.

A total of 120 (11-mm × 11-mm) squares for the four lupin plants and 108 (11-mm × 11-mm) squares for the four soybean plants were defined as ROIs in the root system and the corresponding rhizobox soil imaging data (top of Fig. 5a,b). The relative ^11^C radioactivity of the ROIs was used to generate independent scatter plots between the relative ^11^C radioactivity for the root (*x*-axis) and the soil (*y*-axis) for each white lupin and soybean plant (Fig. 5a,b). The *x*-axis values were calculated from the average of the relative ^11^C radioactivity levels in each ROI during the last 5 min of the root imaging period using the root imaging data in Figs. 3 and S1. Thus, the *x*-axis value indicates the arrived relative ^11^C radioactivity in the underground parts inside the rhizobox within the root imaging period (Fig. 2b, Step 1), i.e., including the ^11^C activity of the roots and soil. The corresponding *y*-axis values were calculated from the average of the relative ^11^C radioactivity levels in each ROI during the first 5 min using the soil imaging data in Figs. 3 and S1. The hotspot blocks (red circles) for white lupin included the entire hotspot area presented in Figs. 4a and S2a, but the other blocks did not (white circles). The correlation between the relative ^11^C radioactivity of the root and soil was determined for the hotspot blocks and for the other blocks, after which the coefficient of determination (R^2^) values were calculated (Fig. 5a). All the square-blocks of the soybean roots and soil were used for analysing correlations and calculating R^2^ (Fig. 5b). To compare the C release capabilities of the whole root system between the two test species, the proportions of ^11^C released from the whole root system to the soil, as the average release rates (%) in the rhizobox areas, were calculated. The average value of the last 5 min of the root imaging period was used to calculate the ^11^C radioactivity of the entire underground part (roots plus soil) and it was defined as the ^11^C radioactivity arrived in whole root system because the ^11^C of soil was released from the root system. The average value of the first 5 min of the soil imaging period was used for the released ^11^C radioactivity levels in the soil. Subsequently, the ratio of the ^11^C radioactivity level of the soil to that of the root was calculated for each test plant (Supplementary Tables 1 and 2).

## Results

### Distribution of ^11^C-photoassimilates in the root system and soil

Several images of ^11^C-photoassimilates partitioning in the root system and the rhizobox soil are shown in Figs. 3 and S1. The ^11^C-photoassimilates translocated from the leaves reached the root base within 20 min after the ^11^CO_2_ feeding and were then gradually delivered to the whole root system in white lupin and soybean plants. The ^11^C-photoassimilates passed almost uniformly through the primary root and then were gradually translocated throughout the entire root system, including the root tips, within 70–90 min in white lupin and 65 min in soybean. In white lupin, the shape of the ^11^C signal in the final images of the root system did not reflect the relatively uniform distribution of the root system. Distinct hotspots of radioactivity occurred in some parts of the root system. An analysis of the distribution of released ^11^C from white lupin into the soil revealed a hotspot with a locally high ^11^C-activity level (Figs. 3a and S1a). In contrast to white lupin, the soybean plants showed almost uniform and coincident distributions of ^11^C in the soil and root system architecture in the final image. The distribution of released ^11^C from soybean in the rhizobox area was comparatively uniform (Figs. 3b and S1b).

### Carbon input into the soil

The densities of ^11^C released from the root differed between white lupin and soybean (Fig. 4). The density of released ^11^C from white lupin was highest in ROI 2, followed by ROI 3, ROI 4, ROI 6, ROI 5, and ROI 1, representing the six hotspots in the rhizobox soil in this case (Fig. 4a). The average density of released relative ^11^C radioactivity in the rhizobox area (mm^2^) was approximately 2.0 Bq MBq^−1^ mm^−2^ in 80 min. The density of the released relative ^11^C radioactivity per hotspot area (mm^2^) was approximately 10.0–24.2 Bq MBq^−1^ mm^−2^ in the six ROIs of the hotspots. Therefore, the release density in the hotspots was greater than the average of the rhizobox area by approximately 5–12 times. In contrast to white lupin, the released ^11^C from soybean was almost uniformly distributed in the rhizobox area that was in contact with roots (Fig. 4b). The density of released relative ^11^C radioactivity was approximately 1.5 Bq MBq^−1^ mm^−2^ in the rhizobox area, and the highest values on both sides of the rhizobox area were approximately 2.9 and 2.6 Bq MBq^−1^ mm^−2^. In four test plants each of white lupin and soybean, the density of released ^11^C in the rhizobox areas ranged from 0.2 to 2.0 Bq MBq^−1^ mm^−2^ and 0.7 to 1.6 Bq MBq^−1^ mm^−2^, respectively, and there was no significant difference between the two species. However, compared with soybean, white lupin released ^11^C in particular areas to form several hotspots, with different values (ranged from 1.0 to 24.2 Bq MBq^−1^ mm^−2^) in different plants (Figs. 4 and S2).

Independent scatter plots between the ^11^C radioactivity of the root and soil for four test plants of white lupin and soybean were prepared (Fig. 5). Each plot represents a square ROI block set in a position corresponding to the relationship between the root system and the soil. The red circles in the white lupin indicate the hotspot blocks that were selected to include the entire hotspot ROIs presented in Figs. 4a and S2a. For both test plant species, the values of the released relative ^11^C radioactivity in the soil (*y*-axis) tended to increase in proportion to the increasing values of the ^11^C radioactivity arrived in the root (*x*-axis). The correlation between the relative ^11^C radioactivity in the soil and the root plus soil was moderate, with R^2^ = 0.519–0.592 (p < 0.0001) for soybean and R^2^ = 0.515–0.677 (p < 0.0001) for white lupin, except for the hotspot blocks. In the hotspot blocks, the relative ^11^C radioactivity value of the soil was not necessarily proportional to the increase in the value in the root. The correlation of the hotspot blocks was lower than that of the other blocks (R^2^ = 0.005–0.695, p < 0.0001 for one plant and p = 0.0426–0.8419 for three plants) for white lupin. In the rhizobox area, the arrived ^11^C radioactivity of the entire underground part was 11–33 and 7–47 MBq for lupin and soybean plants, respectively. In the corresponding soil, the released ^11^C radioactivity was 74–827 and 111–1020 kBq for lupin and soybean plants, respectively (Supplementary Tables 1 and 2). The proportions of released ^11^C from the root to the soil were approximately 0.3%–2.9% for white lupin and 0.9%–2.3% for soybean (Supplementary Table 2). In soybean plants, the value of released ^11^C in each ROI was generally up to twice the average proportion of released ^11^C from the whole root; however, white lupin released a considerable proportion of its ^11^C in hotspot block areas (red circles), and its maximum value was approximately 4.7 times the average proportion of ^11^C released from the other block areas (white circles). In comparison with the released proportion, the maximum ^11^C activity level in hotspot areas was 23.1 times greater than the average ^11^C activity level of the other areas.

## Discussion

In previous studies, various types of rhizoboxes were developed to analyse C allocation and its components in soil-grown plants^5,9,16,33–35^. One of the reasons for investigation of C allocation in belowground being difficult is that it is not easy to separate the root and surrounding soil noninvasively; therefore, the amounts of ^14^C- or ^13^C-assimilates allocated to the roots and surrounding soil could not be differentiated. Our rhizobox system allows rapid separation of the soil and roots without destroying the soil surface. Therefore, the rhizobox system allowed the imaging of a small amount of ^11^C released into the rhizobox soil (Figs. 1 and 2). Compared to other growing conditions (e.g., big rhizoboxes or natural conditions), the small, two-dimensional design of the rhizobox causes the roots to be restricted to a narrow space, which may have limited the C allocation rate into the belowground area. This potential drawback may suggest that the method requires improvement; notwithstanding, the rhizobox can yield a simple form of root system that can be easily observed, and includes the advantage that the method enables quantitative analysis of the spatial distribution of ^11^C-photoassimilates in the root system inside the rhizobox for an intact, living plant (Fig. 3). In contrast to ^14^C-tracers, ^11^C is suitable for the direct visualisation of roots in soil because the energy of the positron annihilation gamma-rays from ^11^C is high (511 keV); therefore, more than 99% of the gamma-rays can permeate the rhizobox (including the plant body and 14.5 mm of the soil box) into each PETIS detector, unlike the beta-rays from ^14^C. To the best of our knowledge, the present method is the first experimental system to successfully visualise and quantify the released C in different root positions of soil-grown living plants. This feature permits the positions of the roots and the surrounding soil to be aligned, enabling the sampling of the soil based on ROIs after the PETIS imaging.

In previous studies, fixed ^14^C was translocated from the leaves to the roots within 1 hour, and the exudation from the roots increased to a maximum after 2–3 hours of fixation in wheat^14^. In soybean plants, the photoassimilates were transported from leaves to the root base within 20 or 30 min after fixation, and the distribution of ^11^C-photoassimilates in the soil-grown root system was visualised^12,13,28^. The present results were consistent with those of previous studies, but they also indicated that photoassimilates translocation for 65–90 min is sufficient for the release and visualisation of the released C in the soil for white lupin and soybean plants. However, there is some difficulty in assessing the C allocation pattern of the underground part during the entirety of plant growth because the half-life of ^11^C is short, meaning that the PETIS imaging must be carried out over a short period of time. Zang *et al.* (2019) reported that the plant age and the chase period strongly affected C allocation patterns. The C allocated belowground (roots and soil) strongly decreased with plant age, but markedly increased within the first 10 days of the chase period after ^14^C or ^13^C labelling in the rice–soil system. Therefore, it is necessary to combine the three tracers (^11^C, ^14^C, and ^13^C) to cover C allocation over both the short and long term to correctly evaluate C allocation patterns as well as the spatial C distribution in roots and soil. Thus, the large variability of the C release proportions among the individual plants (Supplementary Table 2) may have been affected by interactions of these complex factors, such as plant age and the chase period of imaging.

The signals of the ^11^C-photoassimilates translocated into the root system (Figs. 3 and S1) included the signals of the released ^11^C-photoassimilates in the rhizobox soil before the removal of the root system from the rhizobox. However, because the signals of the released ^11^C were much weaker (less than 3%) than the signals of the ^11^C-photoassimilates in roots, it was necessary to separate the signals derived from the plant root from those derived from the released substances in the soil. The recently assimilated C from leaves arrived at the root base approximately 14 min after the tracer feeding in the plant shown in Fig. S3, which was similar to the timing presented in Fig. 3. The release of organic substances into the nutrient solution was first detected approximately 35 min after tracer feeding into the bathing solution using a trap column cartridge filled with active carbon (Fig. S3b right). Therefore, the released ^11^C-photoassimilates may be secreted as organic substances into the surrounding areas approximately 20 min after the photoassimilates are translocated into the root system. Moreover, the ^11^C signal from the root system to the soil included the ^11^C released from 40 to 65–90 min after the tracer feeding (Figs. 3 and S1).

The distribution of the released ^11^C in the rhizobox soil may conceivably include the ^11^C-rhizodeposits and ^11^CO_2_ that originated from root and rhizomicrobial respiration. However, the ^11^CO_2_ produced by respiration was able to dissolve in soil water or diffuse through the nylon mesh into the soil. Thus, the ^11^C signal in the soil may include the ^11^C activity due to respiration. Helal and Sauerbeck (1991) reported that in bean plants, rhizosphere respiration is consumed as 20% of the total assimilated C, and the rhizomicrobial and root respiration contributes 76%–84% and 16%–24% of the total rhizosphere respiration, respectively. In other recent studies on crops and grasses, about 10% and 16% of the assimilated C was allocated to the roots, with about 8% and 12% of the assimilated C lost as root respiration and about 3% and 5% of the assimilated C lost as rhizodeposits, respectively^7^. Therefore, a large amount of ^11^CO_2_ derived from root respiration, representing more than double the total amount of rhizodeposits, could be contained in the image data of the soil area. However, these previous studies estimated respiration based on the total root system or soil over long periods (more than a few hours). In the current study focusing on hotspots and the local areas over a short period, the amount of released ^11^C was 5–12 times higher in hotspots than in other areas (Fig. 4). Additionally, the respiration rate can vary by up to 2 times among the various positions of cluster roots and other root types in white lupin^36^. These findings may suggest that at least in the hotspots of white lupin, the ^11^C signal in the soil is mostly derived from the ^11^C-rhizodeposits released from the local roots. However, because of technical difficulties in separating the ^11^CO_2_ from the soil in our method, our results may need to be validated by new methods in future work. The soil imaging data of soybean were relatively uniform and did not reflect the clear shape of the soybean root system, which may be the result of the following: 1) the signal of the released ^11^C in the soil was too weak and the background noise was amplified; 2) the adjacent roots were intertwined with each other in the nylon-mesh bag, resulting in a mixture of released ^11^C from different roots; and 3) the released ^11^C from the roots was diffused in the water between the roots and the soil. Owing to these factors, the resolution of the released ^11^C imaging data decreased to approximately a few centimetres. One of the limitations of our method is that the resolution of the rhizodeposition imaging data is lower than that of phosphor imaging. Therefore, it may not be suitable for analysing a single root. However, a high released ^11^C signal was detected in white lupin, revealing a definite hotspot in the local soil area. Thus, the above-mentioned problems may be addressed by improving the high signal to noise ratio.

White lupin forms a structure called cluster roots in which lateral roots are densely packed. They secrete large amounts of organic acids and enzymes into the surrounding soil over a short period and intensively take up nutrients, including phosphorus and iron. The amount of root exudation varies by several fold depending on the changing surrounding environment and relationship with the root system’s growth stage at specific positions^1,2,6^. Li *et al.* (2010) reported that the concentration of citrate, a major fraction of released carboxylates, in the surrounding soil of white lupin cluster roots changed from 1.4 to 11.4 µmol g^−1^ dry soil under different soil conditions. In the present study, the released ^11^C distribution from white lupin showed a hotspot, with a very high localised ^11^C concentration, and the presence of cluster roots was confirmed. However, the other cluster roots did not release large amounts of ^11^C to form hotspots. This may have been because of the differences in the cluster root growth stages and/or soil environmental conditions. In contrast to white lupin, soybean does not form cluster roots, resulting in a uniform distribution of released ^11^C. However, the arrival times of ^11^C-photoassimilates into roots and the proportions of released ^11^C for the entire root systems were not significantly different between white lupin and soybean. The present method allowed us to determine the spatial C distribution in both the root system and soil and to better identify characteristic locations, like hotspots, in the rhizobox soil. Thus, from a physiological point of view, the PETIS imaging method can provide novel scientific information. Our results visually clarified for the first time that white lupin and soybean plants have different strategies for the release of ^11^C into the soil. These findings indicate that our method is sufficiently able to characterise the diversity in the release of recently assimilated ^11^C into the surrounding soil from different portions of the root system.

In conclusion, we developed and demonstrated the utility of a novel method for the visualisation and evaluation of the ^11^C-photoassimilate distributions in the root system and the rhizobox soil using white lupin and soybean as test plants. The results suggest that the ratio of photoassimilate allocation in the root and the released ^11^C in the surrounding soil differs spatially in the root system and that the C allocation process varies depending on the plant species. Our method demonstrates the possibility of the non-destructive sampling of roots and surrounding soil at specific positions in the root system based on the PETIS imaging data. It also allows for analyses of the chemical and biological characteristics of the surrounding soil without radionuclides after the PETIS imaging because the half-life of ^11^C (20.39 min) is short. The released C compounds have important effects on microbial populations and nutrient solubility and availability, while also enhancing the plant’s ability to cope with adverse soil–chemical conditions^1,2,37,38^. Therefore, the method will be useful for future investigations of the mechanisms involved in, and the control of, plant secretory systems, the effects of the released C on abiotic and/or biotic factors, and the ecological functions of plant–rhizosphere microbial community interactions.

## Supplementary information

Supplementary information.
