# Supplementary material for: Visualising spatio-temporal distributions of assimilated carbon translocation and release in root systems of leguminous plants
Source: Sci Rep. 2020 Jun 11;10:8446. doi: 10.1038/s41598-020-65668-9 (PMC7289824; doi:10.1038/s41598-020-65668-9)
Supplement: Supplementary file 1 — Supplementary information. [file 41598_2020_65668_MOESM1_ESM.pdf]

Full title:

Visualising spatio-temporal distributions of assimilated carbon translocation and release in root systems of leguminous plants

Yong-Gen Yin[1*], Nobuo Suzui[1], Keisuke Kurita[1,5], Yuta Miyoshi[1], Yusuke Unno[2], Shu Fujimaki[1,6], Takuji Nakamura[3], Takuro Shinano[4,7], and Naoki Kawachi[1]

[1] Takasaki Advanced Radiation Research Institute, National Institutes for Quantum and Radiological Science and Technology (QST), Gunma 370-1292, Japan

[2] Department of Radioecology, Institute for Environmental Sciences, Aomori 039-3212, Japan

[3] Agro-environmental Research Division, NARO Hokkaido Agricultural Research Center, Hokkaido 062-8555, Japan

[4] Agricultural Radiation Research Center, NARO Tohoku Agricultural Research Center, Fukushima 960-2156, Japan

[5] Current address: Materials Sciences Research Center, Japan Atomic Energy Agency, Tokai, Ibaraki 319-1195, Japan

[6] Current address: Institute for Quantum Life Science, National Institutes for Quantum and Radiological Science and Technology, Chiba 263-8555, Japan

[7] Current address: Research Faculty of Agriculture, Hokkaido University, Sapporo, Hokkaido 060-8589, Japan

* Corresponding author: Yong-Gen Yin

E-mail: yin.yonggen@qst.go.jp

Telephone number: +81 27 346 9523

Fax number: +81 27 346 9353

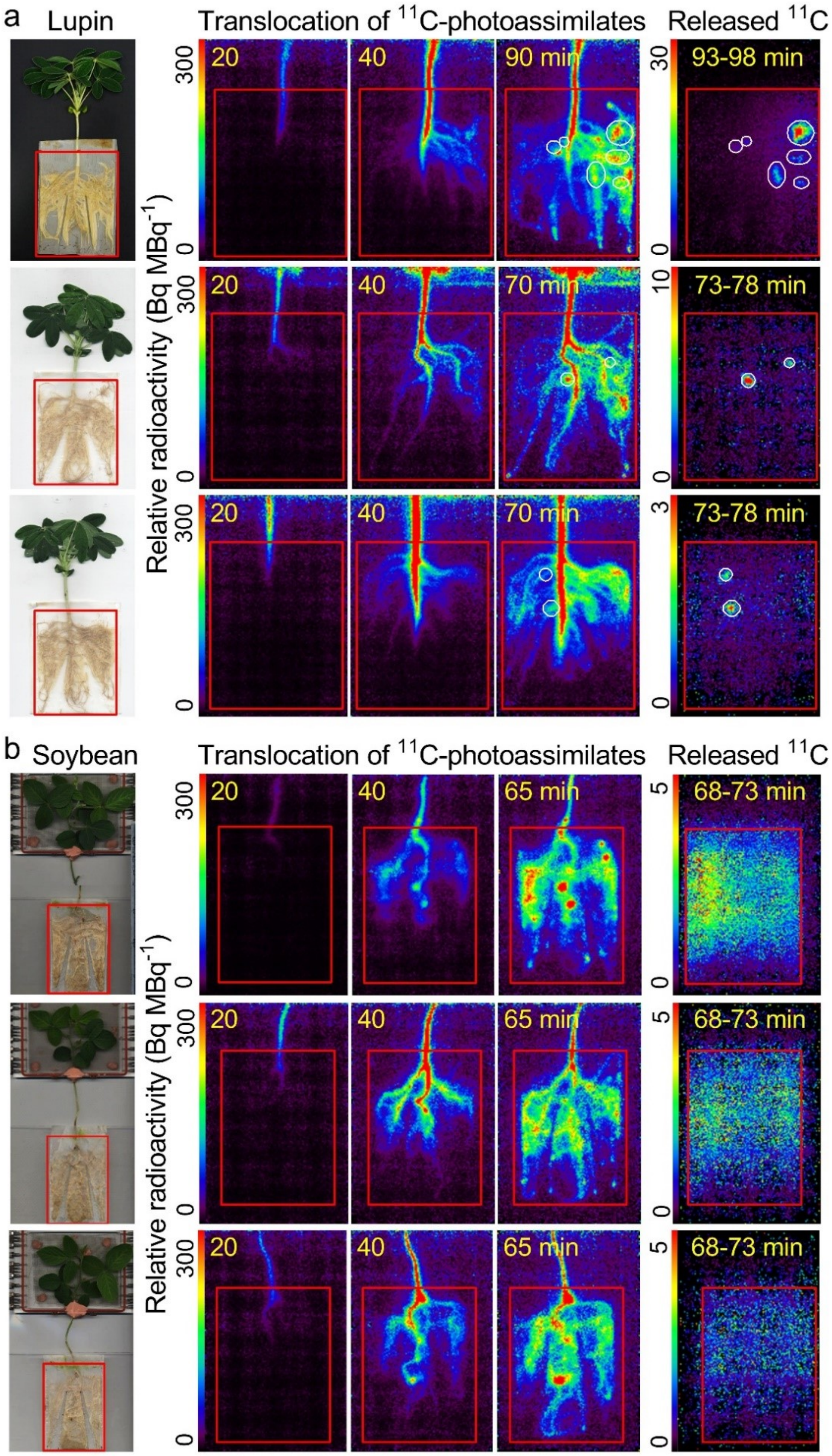

Fig. S1 Repetition of imaging experiments using different white lupin (a) and soybean (b) plants.

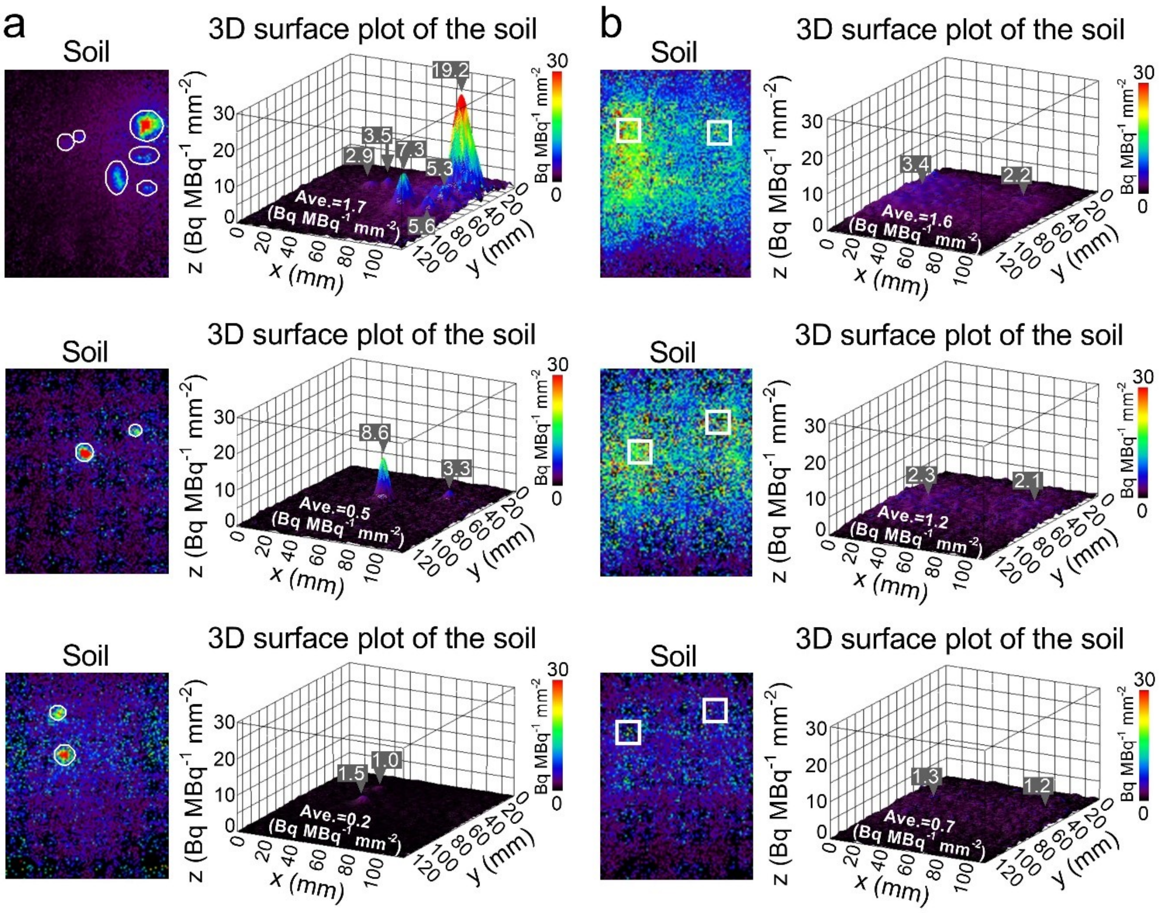

Fig. S2 Repetition of the analysis of the 3-D surface plot data from different test plants of white lupin (a) and soybean (b).

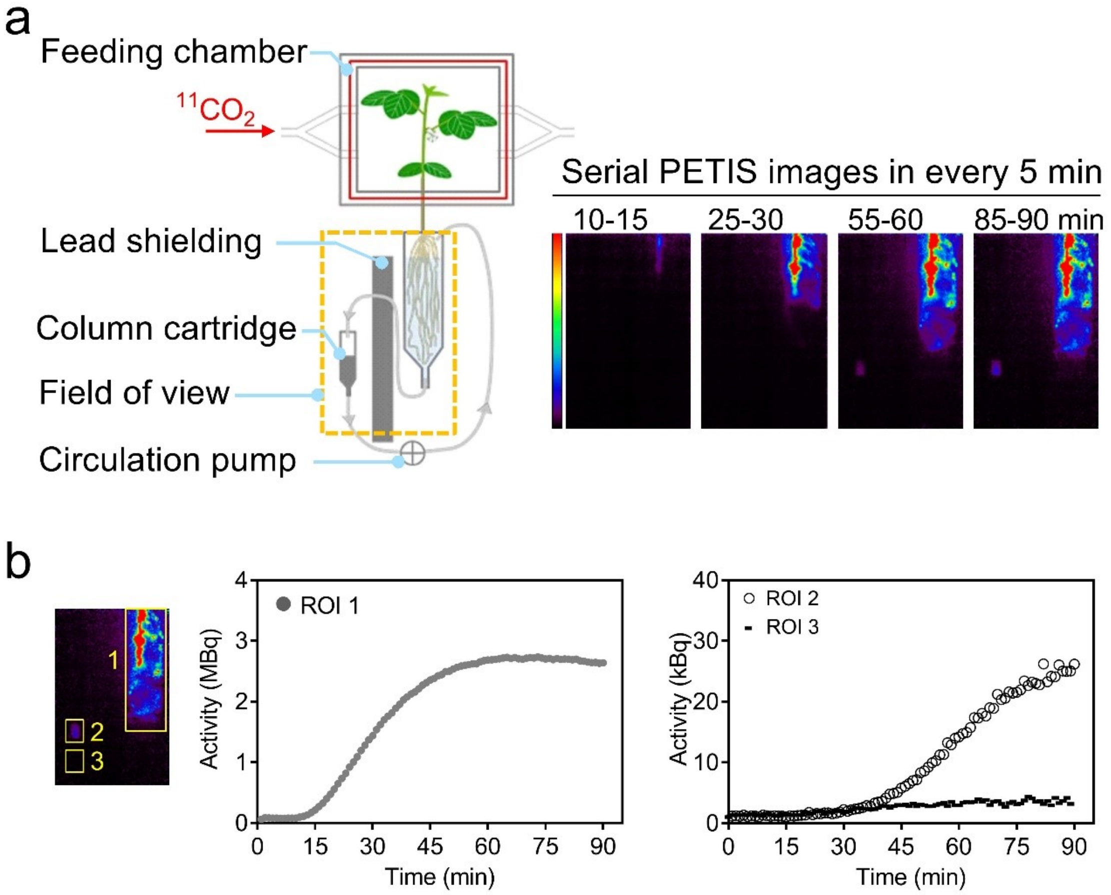

Fig. S3 Imaging data of ^{11}C-labelled photoassimilates translocated into the soybean root and ^{11}C-labelled organic substances released by roots. A 35-d-old soybean plant cultivated in nutrient solution was used in the experiment. (a) Schematic illustration of the arrangement of the imaging experiment using the PETIS and $^{11}CO_2$ (left). The InertSep Active Carbon column cartridge (GL Sciences Inc., Tokyo, Japan) was placed on the left side of the root and shielded with lead. The nutrient solution was circulated by a pump (UPS-112E, NITTO KOHKI CO., LTD., Tokyo, Japan) at 13 ml min^{-1} through the column cartridge to adsorb the organic substances secreted by the root. The aerial part of the plant was fed with a pulse of 180 MBq $^{11}CO_2$ in ambient air constantly supplied by a pump at a rate of 100 ml min^{-1}, and the PETIS was immediately started to obtain images. The root and the column cartridge were positioned just inside the field of view of the PETIS detector heads. The obtained PETIS imaging data (right) visualised the ^{11}C-labelled photoassimilates translocated into the root system and the ^{11}C-labelled organic substances adsorbed by the column cartridge. Each PETIS image is a composite of original images collected every 5 min. (b) Time-course analyses of ^{11}C-labelled photoassimilates generated by the PETIS imaging data. Positions of ROIs 1–3 in the PETIS imaging data (left) were in the root system (ROI 1), the column

cartridge (ROI 2), and background (ROI 3). Time-courses of the ^{11}C activity dynamics in the root (middle) and of the column cartridge and background (right). The *y*-axis values indicate the ^{11}C activity in each ROI after being corrected for the total assimilated ^{11}C-activity levels in leaves.

Supplementary table 1 Provided ^{11}C radioactivity and arrived ^{11}C radioactivity in the rhizobox area (including ^{11}C of the roots and soil) within the root imaging period, and the proportion of arrived to provided radioactivity.

	Plant no.	Feeding radioactivity (MBq)	Arrived ^{11}C in rhizobox (MBq)	Arrived proportions (%)	Average (%)
Lupin	1	200	11	5.5	10.8 ± 4.5
	2	200	33	16.5	
	3	200	20	10.0	
	4	200	22	11.0	
Soybean	1	300	20	6.7	7.0 ± 2.7
	2	450	47	10.4	
	3	190	7	3.7	
	4	220	16	7.3	

Supplementary table 2 Arrived ^{11}C radioactivity and released ^{11}C radioactivity in the rhizobox area, and the proportion of released to arrived radioactivity.

	Plant no.	Arrived ^{11}C in rhizobox (MBq)	Released ^{11}C in soil (kBq)	Released proportions (%)	Average (%)
Lupin	1	11	314	2.9	1.6 ± 1.3
	2	33	827	2.5	
	3	20	153	0.8	
	4	22	74	0.3	
Soybean	1	20	464	2.3	1.8 ± 0.6
	2	47	1020	2.2	
	3	7	111	1.6	
	4	16	151	0.9	
